# Clinicopathological and prognostic evaluations of anorectal cancer after fecal diversion for patients with Crohn’s disease

**DOI:** 10.1186/s12876-021-01751-3

**Published:** 2021-04-13

**Authors:** Hirosuke Kuroki, Akira Sugita, Kazutaka Koganei, Kenji Tatsumi, Ryo Futatsuki, Eiichi Nakao, Nao Obara, Katsuhiko Arai

**Affiliations:** Department of Surgery for Inflammatory Bowel Disease, Yokohama Municipal Citizen’s Hospital, 1-1, Mitsuzawanishicho Kanagawa-ku, Yokohama City, 221-0855 Japan

**Keywords:** Crohn's disease, Fecal diversion, Anorectal cancer, Abdominoperineal resection, Surveillance, Stoma

## Abstract

**Purpose:**

Colorectum diversion with a proximal stoma is often the preferred surgical approach in patients with Crohn's disease-related anorectal lesions or refractory colitis. To date, few studies have assessed the incidence and prognosis of cancer in the diverted anorectal segments. This study aimed to evaluate the clinical characteristics and prognosis of anorectal cancer associated with Crohn's disease following fecal diversion.

**Methods:**

This was a retrospective study based on medical records of patients diagnosed with Crohn’s disease between 1999 and 2020. It was conducted at Yokohama Municipal Citizen’s Hospital. Patients diagnosed with anorectal cancer following fecal diversion were identified, and their prognosis was the primary outcome measure.

**Results:**

Among 1615 patients, 232 patients (14%) underwent colorectum diversion. Of those 232 patients, 11 were diagnosed with anorectal cancer following fecal diversion, ten were diagnosed with advanced cancer, 10 underwent abdominoperineal resection, and eight died. 1 could not undergo resection due to multiple lung metastasis and died. The overall five-year survival rate in patients diagnosed with anorectal cancer following fecal diversion was 20%.

**Conclusion:**

Crohn's disease-associated anorectal cancer following fecal diversion was challenging to diagnose early, and patients had a poor prognosis even after curative resection. Early abdominoperineal resection may be considered for patients with Crohn's disease who cannot benefit from cancer screening and surveillance due to difficulty accessing the anorectal stricture via endoscopy.

## Introduction

Crohn's disease (CD)-related anorectal cancer following fecal diversion is a rare disease. Although the risk of colorectal cancer (CRC) has been well described, the risk of CRC in diverted bowel segments is lacking, and the current risk remains unclear [1]. Typically, CD-associated cancers present in advanced stages are poorly differentiated and have a worse prognosis than usual adenocarcinoma [2–5]. For this reason, surveillance colonoscopy is necessary to detect cancer at an early stage. For cancer surveillance in patients with CD, periodic colonoscopy, and histopathological examination of dysplasia is considered useful in western countries [6–8]. European Crohn's and Colitis Organization guidelines suggest performing a colonoscopy every 2–3 years in intermediate-risk patients, defined as patients with extensive colitis and mild to moderate active inflammation, the presence of post-inflammatory polyps, or CRC in an immediate family member > 50 years of age. Low-risk patients without risk factors may have screening colonoscopies performed every 5 years [9]. Conversely, there are no consensus guidelines regarding clinicopathological findings and surveillance biopsies for diverted segments. However, when a stoma is created for severe anorectal lesions or when refractory colitis or anal proctitis is diverted in CD patients, inflammatory stricture occurs in the diseased lesion. This inflammatory stricture makes it challenging to investigate the diverted anorectum using endoscopy. Therefore, if cancer develops in the excluded part of the anorectum, it is usually diagnosed at a more advanced stage than usual CD-related anorectal cancer (ARC), resulting in a poor outcome. This study aimed to clarify the clinicopathological course and outcomes of ARC following fecal diversion in patients with CD.

## Materials and methods

### Patient selection

This retrospective single-institution study was conducted to evaluate the occurrence, clinicopathological characteristics, risk factors, and prognosis of CD-associated anorectal cancer following fecal diversion (diverted anorectal cancer). Before initiating this study, institutional approval was obtained from the ethical Advisory Committee of Yokohama Municipal Citizen’s Hospital. Data of consecutive CD patients with diverted ARC, treated between January 1999 and 2020, were evaluated. The characteristics, clinical course, and follow-up were reviewed from our institutional database and individual chart reviews. A prolonged diversion was defined as diversion > 24 months.

The data set included sex, age at diagnosis of CD, site, extent of CD, duration between diagnosis of CD and stoma creation, indication of stoma (anorectal lesion vs. refractory colitis), site of stoma (ileo-/jejuno-stomy vs. colostomy), type of stoma (loop vs. end [Hartmann's procedure]), the continuous symptom of colorectum diversion, duration of CD, duration of fecal diversion; smoking history; biologics, immunomodulator administration; surveillance biopsy; tumor site; cancer diagnosis; maximum tumor size; TNM staging; recurrence; and histological type of cancer.

ARC was defined according to the location of the diverted anorectum or perianal fistula and classified and staged according to the 8th Union for International Cancer Control (UICC) pathological TNM staging system. Radial margin positivity was defined as having a negative margin of < 1 mm in the resected specimens.

### Perioperative management and surgical procedure

Perioperative medical treatments for CD lesions follow the Ministry of Health, Labor, and Welfare guidelines in Japan. The standard medical treatments include 5-aminosalicylic acid, prednisolone, immunomodulator, and biologics for CD-associated lesions without stricture and infection. Cancer surveillance was performed only upon receipt of the patient's written consent. However, most patients reject the colonoscope due to pain. Therefore, we performed cancer surveillance if patients had symptoms, such as anal pain, increased discharge, and bleeding. The surveillance method was a colonoscopic biopsy, mucin cytology, and examination under anesthesia (EUA). ARC may be diagnosed during surgery with other CD-related indications or from resected specimen's pathological findings.

One of the standard surgical procedures for a CD with the severe colorectal disease was fecal diversion (the creation of loop stoma or Hartmann's procedure). Many Japanese patients tend to select fecal diversion to preserve the anus and close the stoma. Younger patients also tend to avoid abdominoperineal resection (APR) and total proctocolectomy (TPC) because of possible sexual and urinary dysfunction. Most loop stomas are created in the patients' ileum except for those with a short residual small intestine. Hartmann's procedure is performed for localized, severe anorectal lesions. However, if the lesions worsen, for example, continuous pus discharge from multiple anal fistulae, remnant-colorectum continuous mucous discharge, or anal pain, we perform APR or TPC. If ARC occurs, we perform APR or TPC. Preoperative chemoradiotherapy is not usually performed for ARC due to possible infection.

We performed regular follow-up examinations every two weeks up to three months after APR or TPC at our outpatient center. Follow-up examinations (examining tumor markers [carcinoembryonic antigen and cancer antigen 19–9] and computed tomography) were performed every 6 months. Time to follow-up was measured as the time from APR or TPC to the most recent clinical follow-up or death. Follow-up examinations were performed until July 31, 2020.

### Outcomes

The primary outcome was defined by the incidence of diverted ARC. Possible risk factors for diverted ARC included sex, extent of CD (ileocolitis vs. colitis), duration between diagnosis of CD and stoma creation, indication of stoma (anorectal lesion vs. refractory colitis), site of stoma (ileo-/jejuno-stomy vs. colostomy), type of stoma (loop vs. end [Hartmann's procedure]), the continuous symptom of colorectum diversion, biologic administration (infliximab and adalimumab), duration of CD, and duration of fecal diversion.

The secondary outcome was the comparison of diverted and non-diverted ARC.

The tertiary outcome was the comparison of cumulative survival rate after APR between diverted and non-diverted ARC.

### Statistical analysis

Continuous variables were compared using the Mann–Whitney *U* test, and the results are expressed as the cut-off value in the ROC curve analysis. The cut-off values for the duration from diagnosis to stoma creation were defined as the values nearest to the upper left corner of the analyses in this series (205 months). Odds ratio (OR) and 95% confidence intervals (CI) were calculated for all variables in univariate analysis. Each factor with a significant *p* value in the univariate analysis was entered into a stepwise logistic regression model. The data were presented as the median and range. The level of statistical significance was set at *p* < 0.05. After fecal diversion was estimated using the Kaplan–Meier method, the overall survival after diagnosis of ARC was estimated, and the log-rank test evaluated differences between curves. All statistical analyses were performed using the R statistical computing software.

## Results

### Patients’ characteristics in diverted ARC

**Fig. 1:**
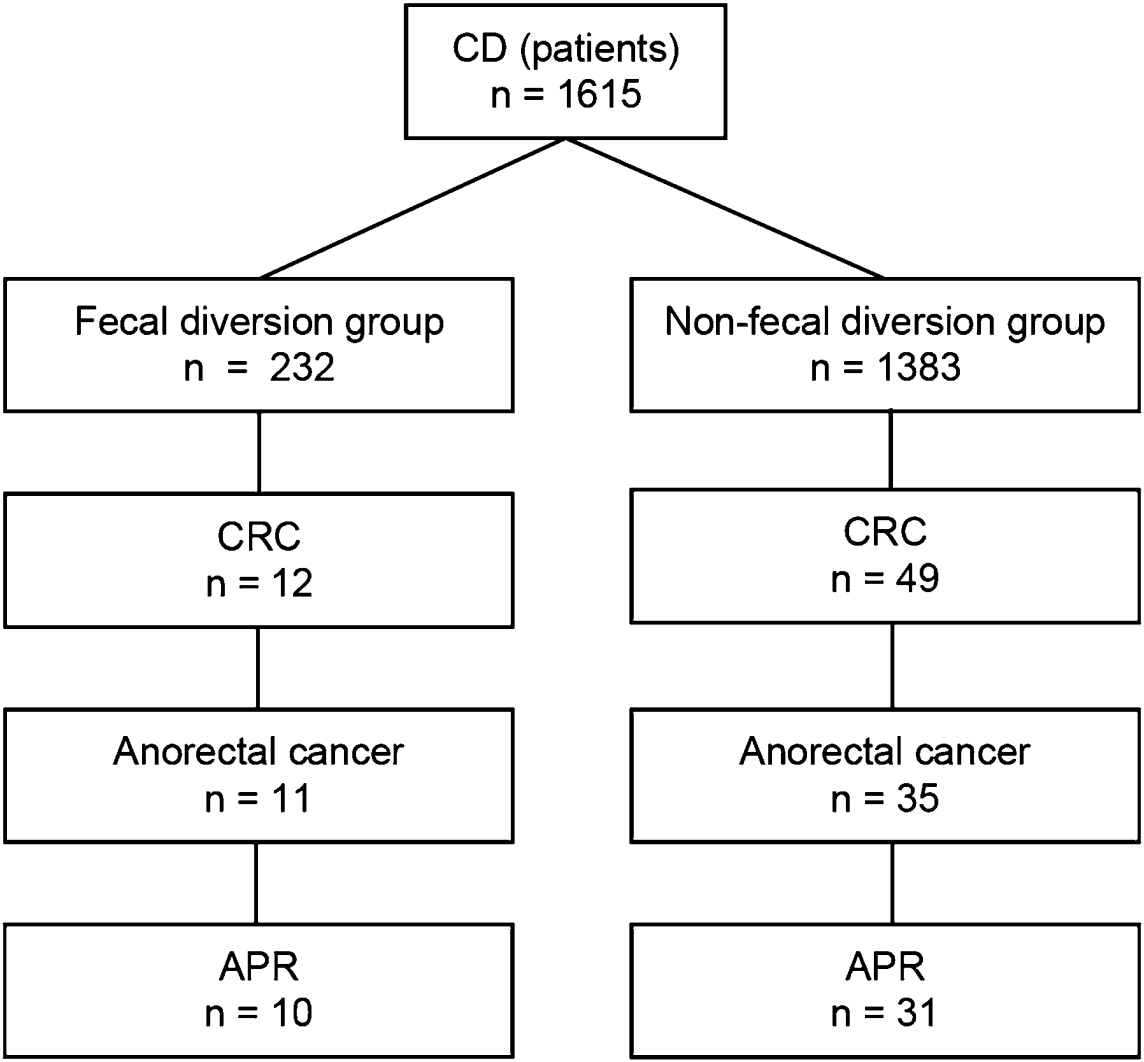
Patient selection. *APR* abdominoperineal resection, *CD* Crohn's disease, *CRC* colorectal cancer

There were 232/1,615 (14.3%) patients that underwent fecal diversion, and 11 of those (4.7%) patients had anorectal cancer (Fig. 1). Of the 11 patients, 10 underwent APR and diverted ARC was pathologically diagnosed in the original surgical specimen and one patient could not be operated because the tumor was unresectable with multiple lung metastasis. The 10 patients with diverted ARC were compared with 31 patients with non-diverted anorectal ARC.

The characteristics of the 10 patients are presented in Table 1. Six of the ten patients were female, and the median age at CD anorectal cancer diagnosis was 18 (range 9–30) years. Eight patients had ileocolitis, and eight had perforating diseases. Eight patients were smokers, two were treated with biologics, and two with immunomodulators. Only one patient underwent an annual surveillance biopsy. A total of seven (70%) patients had anorectal cancer. The remaining three (30%) patients had cancer arising from the perianal fistula. In cancer diagnosis, seven patients were diagnosed with cancer before the surgery, two patients were diagnosed during the surgery, and one patient was diagnosed with cancer from a resected specimen after surgery. The median tumor size was 65 mm. In terms of depth invasion, nine (90%) patients were T3–T4, and four (40%) patients had lymph node metastasis. Two (20%) patients had distant metastasis at the diagnosis time (peritoneum, n = 1; lungs and liver, n = 1). Eight (80%) patients were diagnosed with a mucinous component using resected specimens, and six (60%) patients with a positive radial margin. Nine (90%) patients had either recurrent or remnant cancer. Nine patients (90%; excluding the stage I patient) received chemotherapy. However, nine (90%) patients had a recurrence, and 8 out of 10 (80%) patients died. Table 2 summarize the details of 10 patients diagnosed with diverted anorectal cancer.

**Table 1:** Demographic and clinical characteristics of patients diagnosed with anorectal cancer after fecal diversion

Patient	Sex	Time from CD to stoma creation (months)	Method of stoma creation	Time from stoma to cancer diagnosis (years)	Symptoms	Diagnosis	Location of cancer	Type of histology
1	Female	36	End	31	Anal pain, mucous discharge	Endoscopic biopsy	Anorectal	muc
2	Female	94	End	2	Anal pain	Intraoperative biopsy and cytology	Anorectal	muc
3	Male	248	End	3	Anal pain, bleeding	Cytology	Anorectal	muc, sig
4	Male	144	Loop	8	Bleeding	Endoscopic biopsy	Anorectal	w/d, m/d
5	Male	202	Loop	9	Poor defecation	Intraoperative biopsy	Anorectal	por, w/d, and m/d
6	Male	205	Loop	4	Fever, pelvic abscess	Intraoperative cytology	Perianal fistula	muc, sig
7	Female	273	Loop	2	Anal pain	Endoscopic biopsy	Perianal fistula	tub2
8	Male	143	End	7	Mucous discharge	Biopsy, cytology	Anorectal	muc, sig, and w/d
9	Female	350	Loop	3	Anal pain	Biopsy	Perianal fistula	muc
10	Female	169	Loop	6	Mucous discharge	Resected specimen	Perianal fistula	muc, sig, and w/d

*CD* Crohn's disease, *m/d* moderately differentiated adenocarcinoma, *muc* mucinous adenocarcinoma, *por* poorly differentiated adenocarcinoma, *sig* signet ring cell carcinoma, *w/d* well-differentiated adenocarcinoma

**Table 2:** Outcomes in patients with diverted anorectal cancer after APR

Patient	Resected margin	Type of histology	TNM classification	Chemoradiation	Site of recurrence	Outcome after cancer diagnosis
1	Negative	muc	Stage II	Chemotherapy	Intrapelvic, uterus	Dead at 32 months
2	Negative	muc	Stage II	Chemoradiation	Intrapelvic, inguinal LN	Dead at 45 months
3	Negative	muc, sig	Stage IV	Chemotherapy	Peritoneal dissemination	Dead at 26 months
4	Negative	w/d, m/d	Stage I	Nothing	NED	Alive at 60 months
5	Negative	por, w/d, and m/d	Stage IV	Chemotherapy	Intrapelvic, peritoneal dissemination	Dead at 27 months
6	Positive	muc, sig	Stage III	Chemotherapy	Intrapelvic, bone	Dead at 20 months
7	Negative	m/d	Stage II	Chemoradiation	Intrapelvic, bone	Dead at 36 months
8	Negative	muc, sig, and w/d	Stage II	Chemotherapy	Intrapelvic, paraaortic LN	Dead at 30 months
9	Positive	muc	Stage II	Chemoradiation, heavy ion radiotherapy	Intrapelvic	Alive at 110 months
10	Positive	muc, sig, and w/d	Stage II	Chemotherapy	Intrapelvic	Dead at 3 months

*APR* abdominoperineal resection, *LN* lymph node, m/d moderately differentiated adenocarcinoma, *muc* mucinous adenocarcinoma, *NED* no evidence of disease, *por* poorly differentiated adenocarcinoma, *sig* signet ring, *w/d* well-differentiated adenocarcinoma

### Comparison between CD-associated diverted anorectal cancer and diverted non-anorectal cancer and risk factors for CD-associated diverted ARC

The charts of 1615 patients with operative CD were reviewed during the study period. 45 patients (3.6%) were diagnosed with anorectal cancer. Of those, four patients could not undergo APR because of invasions to other organs. In total, 232 patients underwent stoma creation with the diversion of the colorectum to treat the lesion caused by CD; of these, 28 patients with < 2-year follow-up were excluded. Of 204 patients 140 were males; the median age at the time of stoma creation was 32 years. A total of 11 (5.3%) consecutive patients (5 males, 6 females) with diverted ARP were analyzed. The median follow-up period was 93 months (range 24–382) for the initial stoma creation in the 204 patients (Table 3).

**Table 3:** Patient characteristics at the time of stoma creation

	Overall n = 204	Diverted ARC	Non-diverted ARC	*p* value
Sex (male/female)	140/64	5/6	135/58	0.10
Onset age of CD (years)	19 (1–63)	18 (3–31)	19 (1–63)	0.13
Extent of CD (ileocolitis/colitis)	195/9	9/2	186/7	0.43
Age at stoma creation (years)	32 (10–69)	35 (24–47)	32 (10–69)	0.85
Duration from diagnosis to stoma (months)	142 (2–462)	227 (36–350)	138 (2–462)	0.07
Indication of stoma (anorectal lesion/refractory colitis)	158/46	10/1	148/45	0.29
Site of stoma (ileo(jejuno)stomy/colostomy)	112/92	4/7	108/85	0.21
Type of stoma (loop/end)	102/102	7/4	95/98	0.35
Continuous symptom after stoma creation	57 (27.9)	1 (9.0)	56 (29.5)	0.18
Biologic administration	17 (8.3)	2 (18.1)	15 (8.8)	0.24
Surveillance biopsy for diverted intestinal tract	23 (11.2)	1 (9.0)	21 (10.8)	0.88
CD duration (months)	138 (34–417)	85 (42–417)	143(34–378)	0.20
Duration of fecal diversion (months)	98 (24–382)	46 (24–382)	104 (24–346)	0.21

Data are numbers with percentages in parentheses, unless otherwise indicated. Continuous variables are indicated as median (range)

*CD* Crohn's disease, *ARC* anorectal cancer

Among the 204 patients, 102 had loop stomas. After the initial stoma creation, 57 patients experienced ongoing anorectal symptoms. However, in the diverted ARC group, only 1 patient had anorectal symptoms, and the other 10 patients had improvements.

Table 4 shows no significant differences in the sex, diagnostic age of CD, an indication of stoma, type of stoma, continuous symptoms after stoma creation, biologic administration, duration of CD or duration of fecal diversion between the diverted ARC and diverted non-ARC groups in the univariate and multivariate analysis.

**Table 4:** Logistic regression analysis of the risk factors for CRC in diverted intestinal tract

Factors	Univariate analysis		Multivariate analysis	
	OR (95% CI)	*p* value	OR (95% CI)	*p* value
Male	2.79 (0.81–9.51)	0.10	3.15 (0.78–12.77)	0.10
Type of CD (ileocolitis)	2.31 (0.26–20.33)	0.44	1.46 (0.12–17.42)	0.76
Duration from diagnosis to stoma creation ≧ 205 (months)	1.00 (0.90–1.01)	0.07	6.85 (1.76–26.72)	0.17
Indication of stoma (anorectal lesion)	0.32 (0.04–2.63)	0.29	0.25(0.02–2.40)	0.23
Site of stoma (ileostomy or jejunostomy)	2.22 (0.63–7.84)	0.21	2.56 (0.62–10.64)	0.19
Type of stoma (loop stoma)	1.80 (0.51–6.36)	0.35	3.27 (0.79–13.57)	0.10
Continuous symptom after stoma creation	0.24 (0.03–1.95)	0.18	0.16 (0.01–1.47)	0.10
Biologic administration	2.63 (0.52–13.3)	0.33	2.12 (0.29–15.03)	0.45
Duration of CD ≧ 77 (months)	0.99 (0.98–1.00)	0.20	0.96 (0.89–1.04)	0.43
Duration of fecal diversion ≧ 65 (months)	0.99(0.98–1.00)	0.21	1.02 (0.94–1.10)	0.51

*CD* Crohn's disease, *CI* confidence interval, *ARC* anorectal cancer, *OR* odds ratio

### Comparison of diverted and non-diverted ARC

The two groups were comparable in the characteristics used for matching (Table 5). Site and type of CD, frequency of surveillance biopsy, tumor site and size, mucinous component, and UICC TNM staging were similar between the groups. However, the proportion of recurrence or remnant cancer in patients with diverted ARC was significantly higher than in patients with non-diverted cancer. The use of biologics was higher in patients with non-diverted ARC than in patients with diverted anorectal cancer, but the difference was not statistically significant.

**Table 5:** Patient characteristics

	Diverted anorectal cancer (n = 10)	Non-diverted anorectal cancer (n = 31)	*p*
Sex, male/female	5/5	8/23	0.229
Age at diagnosis with CD (years)	18 (9–30)	23 (11–47)	0.098
Site of CD, colic/ileocolic	2/8	4/27	0.585
Type of CD perforative, n (%)	8 (80)	25 (80.6)	0.865
Smoking, n (%)	3 (30)	8 (38)	0.797
Biologic administration, n (%)	2 (20)	17 (54.8)	0.057
Immunomodulator administration, n (%)	2 (20)	12 (38.7)	0.283
Surveillance biopsy for anorectal lesion, n (%)	1 (10)	3 (9.6)	1
*Tumor site, n (%)*			0.953
Anorectum	7(70)	22(71)	
Perianal	3(30)	9(29)	
*Cancer diagnosis*			0.237
Preoperative biopsy	7	27	
During operation	2	2	
Resected specimen	1	2	
Maximum tumor size (mm)	65 (12–130)	51 (8–85)	0.148
*T category*			0.185
0/1/2/3/4	0/1/0/4/5	1/4/4/12/10	
*N category*			0.453
0/1/2	6/2/2	22/3/6	
Lymphatic vessel invasion ±	5/5	9/22	0.226
Vascular invasion ±	6/4	11/20	0.187
muc component ±	8/2	18/13	0.256
por component ±	4/6	9/22	0.093
Radial margin ±	6/4	15/16	0.588
*UICC TNM stage*			0.105
0/I/II/III/IV	0/1/5/2/2	1/7/14/9/ 0	
Recurrence or remnant of cancer ±	9/1	10/21	0.003*
Observation time after APR (months)	28 (12–151)	47 (1–216)	

Continuous variables are indicated as median (range)

*APR* abdominoperineal resection, *CD* Crohn's disease, *muc* mucinous adenocarcinoma, *por* poorly differentiated adenocarcinoma, *sig* signet ring cell carcinoma

### Comparison of cumulative survival rate after APR

**Fig. 2:**
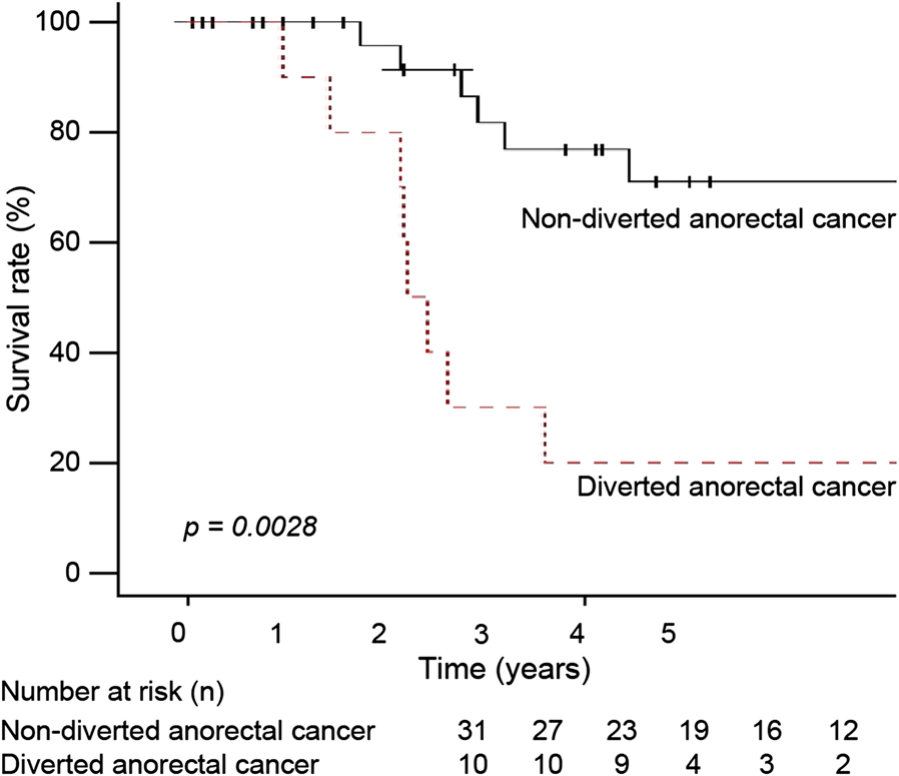
Overall survival in patients after diagnosis of diverted anorectal cancer and non-diverted anorectal cancer. The Kaplan–Meier survival curves are shown (n = 10)

The total 5-year cumulative survival rate after APR was 20% in the diverted anorectal cancer group and 70% in the non-diverted anorectal cancer group (Fig. 2); this difference was significant (*p* = 0.002).

## Discussion

CD-associated diverted CRC is a rare tumor. Carcinogenic rates in previous studies were 0.63% [10] and 3.1% [11]. The meta-analysis reported a relative morbidity risk for CD-associated CRC of 2.5 (range 1.3–4.7) [12], 4.5 (range 1.3–14.9) for large intestinal lesions, and 1.1 (range 0.8–1.5) for ileal lesions. The relative morbidity risk of CRC was 2.4 in a meta-analysis of 60,122 patients with CD reported by Alexander et al. [13]. CD was considered a high-risk factor for cancer [14]. Besides, the following conditions have been reported as characteristics of CD-associated CRC: patients were approximately 10 years younger than those with typical CRC, had a long duration of CD (10–20 years), in many cases, cancer developed in the right colon, the histologic type was mucinous adenocarcinoma, and cancer was likely to develop in lesions caused by CD [15–17]. Bettner et al. reported that while the risk of CRC in inflammatory bowel disease (IBD) populations have been well described, there is a lack of data on the risk of CRC in diverted IBD bowel segments [1]. We believe that our cases can be included in this category. Few studies focusing on diverted CRC have been published, and based on these reports, only a small percentage of cases have been identified [1, 18–22]. These are summarized in Table 6.

**Table 6:** Previous reports on diverted colorectal adenocarcinoma in patients with Crohn's disease

Patient	Author and year	Age, sex	Stoma to cancer (years)	Biopsy before surgery	Histology	Stage	Treatment	Recurrence	Outcome
1	Bettner 2018 [1]	41 M	7	N/A	LGD	N/A	APR		Alive at 11 years
2	Ogawa 2013 [20]	68 F		N/A	muc	IV (TxNxMx)	Not resected		Dead at 5 months
3	Ogawa 2013 [20]	34 F		No malignancy	muc	III (T4N2M0)	APR	No	Alive at 63 months
4	Ogawa 2013 [20]	33 F		Adenocarcinoma	muc, sig	III (T4N0M0)	Pelvic exenteration	Distant	Dead at 14 months
5	Iesalnieks 2010 [22]				muc		APR		Unknown
6	Cirincione 2000 [19]	36 F	2	Squamous cell carcinoma diagnosed at post-surgery	SCC		APR	distant	Alive at 24 months
7	Cirincione 2000 [19]	35 F	9	Adenocarcinoma	por		APR	Distant	Dead at 36 months
8	Cirincione 2000 [19]	54 M	3	Adenocarcinoma	m/d		APR	Pelvis	Alive
9	Church 1984 [18]	65 F		Squamous cell carcinoma	SCC		Radiotherapy	Inguinal lymph node	Dead

*APR* abdominoperineal resection, *F* Female*, LGD* low-grade dysplasia, *N/A* not available, *muc* mucinous adenocarcinoma, *m/d* moderately differentiated adenocarcinoma, *M* Male, *por* poorly differentiated adenocarcinoma, *SCC* squamous cell carcinoma, *sig* signet ring cell carcinoma

None of the references reported on a comparison between the diverted and non-diverted ARC or CD-associated diverted and non-diverted ARC. In our study, there were no significant differences in clinical features and histological findings between the diverted and non-diverted anorectal cancer groups. However, the recurrence and overall survival rates were significantly different. Two cases had stage IV cancer in the diverted anorectal cancer group, although this may be because the diagnosis was made after a very advanced stage, and early diagnosis was required.

Basseri et al. [23] argued that, although there was a strong link between IBD and CRC, an active surveillance colonoscopy approach remained controversial. The Crohn's and Colitis Foundation of America guidelines recommend that at least one-third of the colon 8–10 years after the onset of CD be observed for colonoscopy screening. If surveillance colonoscopy is negative for dysplasia or cancer, an examination is recommended every 1–2 years after that [24]. Currently, there are no consensus guidelines to inform surveillance endoscopy of diverted segments [1]. The insertion of an endoscope was impossible due to the stricture of the diverted large intestine, except for one patient diagnosed at stage I. Pelvic magnetic resonance imaging and EUA are performed in patients who are not colonoscopy candidates due to stricture. However, Devon et al. suggested that cancer might not be diagnosed even after multiple examinations [21]. Sjodahl et al. reported that patients with a diverted colon and rectum with a rectal stump and chronic anal fistula should be under careful surveillance due to their significantly increased risk of cancer [25]. One patient at stage I underwent an annual surveillance colonoscopy and showed disease-free survival. Thus, surveillance colonoscopy or EUA is essential for early-stage cancer diagnosis. The establishment of better surveillance methods for similar cases is urgently needed.

Biologics plays a vital role in managing the active CD. However, biologics are not useful for abscess-forming CD lesions, including complex anal or rectal fistula and rectal stricture. In this study, most patients were not treated with biologics before diversion. We did not use biologics after diversion because there was no evidence of sufficiency. Infliximab was administered to only two patients before surgery. The administration of drugs such as biologics for cancer is controversial because they are associated with immunosuppression [26, 27]. For instance, anti-tumor necrosis factor (TNF) treatment without an immunomodulator does not increase cancer risk in patients with IBD [28]. Investigation of cancer progression associated with treatment using biologics is necessary in most cases. After diagnosing cancer, patients with malignancies generally avoid the administration of anti-TNFα antibody agents because tumor growth may be enhanced by TNFα suppression [28]. The right timing for the administration of biologics after cancer healing must be addressed in the future.

Diverted ARC is generally difficult to treat; surgical resection is the only cure. Diagnoses are typically made at the advanced stage of cancer. In this study, nine patients were diagnosed with advanced cancer (pathological stage II-IV). The prognosis was poor. There was a discrepancy in the staging of clinical and pathological diagnosis; therefore, a negative resected margin was needed. CD-associated with CRC in Japanese patients develops primarily in the rectum and anus. However, the etiology remains unknown. It was challenging to dissect the total mesorectal excision plane because of perirectal inflammation. Recurrence, including intrapelvic recurrence, frequently occurs despite chemotherapy administration after APR. A multidisciplinary treatment combining neoadjuvant chemotherapy, total pelvic exenteration, lateral lymph node dissection (a unique Japanese therapy) during surgery, and perioperative irradiation may be necessary. A previous study (Table 6) reported that four out of nine patients died, showing poor outcomes when anorectal strictures complicate this disease [18–20, 22]. Although there was no evidence for diverted anorectum, the literature suggests that carcinoma may still develop despite perianal healing. Cirincione et al. [19] suggested that surveillance proctoscopy must be performed; otherwise, a preventive APR with permanent fecal diversion should be considered in patients with CD complicated by an anorectal stricture [19]. APR should be performed early to ensure further investigation and prevention of cancer development [22].

For the diversion of anorectum with stricture or inflammation in patients that pose difficulty for screening and surveillance, an early APR may be considered a treatment option that could be done in advance before the patients are diagnosed with advanced cancer based on the symptoms only. A report by van Overstraeten et al. suggested that patients with anorectal CD who need proctectomy should undergo proctocolectomy with ileostomy despite the absence of proximal colonic involvement [29]. Therefore, we opted for APR. These patients passed away from intrapelvic dissemination. Most of the patients with diverted anorectal cancer developed local recurrence. In Europe and the United States, preoperative radiotherapy is a mainstay treatment for rectal cancer, and tumors are often reduced. Japanese standardized treatment for rectal cancer (especially low rectal cancer with T3 depth) is total mesorectal excision with lateral lymph node dissection; however, some CRC research institutions in Japan perform neoadjuvant chemoradiotherapy and report good outcomes [30]. Therefore, in Japan, reducing tumors by preoperative treatment (neoadjuvant chemoradiotherapy) for similar cases should be considered for anorectal cancer. However, the biggest problem is the inability to perform curable surgery because of an intraoperative or postoperative diagnosis, which needs further study.

This study has some limitations. First, it was a retrospective single-institution study and included a limited number of patients. CD therapy in this cohort did not include many patients on biologics or immunomodulator therapy. There are also differences in the treatment strategies for CRCs between Japan, Europe, and America. Second, heterogeneous treatments did not include the use of chemoradiotherapy.

In conclusion, CD-associated anorectal cancer was diagnosed in 11 of 232 (4.7%) patients following fecal diversion, had a poor prognosis after curative resection, and was challenging to diagnose at an early stage. Subsequently, the risk of patients with a diverted CD of the rectum and anus developing cancer cannot be ignored. We recommend annual surveillance colonoscopy or EUA to detect early-stage cancer. In the patients who find it challenging to undergo these modalities, the alternative treatment considered with sufficient informed consent is early APR or TPC to prevent advanced cancer. Thus, further prospective multi-institutional studies with a large population are needed to confirm CD-associated anorectal cancer prognosis following diversion.

## Data Availability

The data underlying this article are available from the corresponding author on reasonable request.
